# Association of Enterotoxigenic *Bacteroides fragilis* with Immune Modulation in Colorectal Cancer Liver Metastasis

**DOI:** 10.3390/cancers17172733

**Published:** 2025-08-22

**Authors:** Rumiko Saito, Yasuyuki Shigematsu, Mahmut Amori, Gulanbar Amori, Manabu Takamatsu, Kenji Nishida, Hiroaki Kanda, Yu Takahashi, Yuji Miura, Kengo Takeuchi, Shunji Takahashi, Kentaro Inamura

**Affiliations:** 1 Department of Medical Oncology, Cancer Institute Hospital, Japanese Foundation for Cancer Research (JFCR), 3-8-31 Ariake, Koto-ku, Tokyo 135-8550, Japan; rumiko.saito@jfcr.or.jp (R.S.); yuji.miura@jfcr.or.jp (Y.M.); s.takahashi-chemotherapy@jfcr.or.jp (S.T.); 2 Department of Clinical Chemotherapy, Cancer Chemotherapy Center, Japanese Foundation for Cancer Research (JFCR), 3-8-31 Ariake, Koto-ku, Tokyo 135-8550, Japan; 3 Department of Pathology, Cancer Institute Hospital, Japanese Foundation for Cancer Research (JFCR), 3-8-31 Ariake, Koto-ku, Tokyo 135-8550, Japan; yasuyuki.shigematsu@jfcr.or.jp (Y.S.); amori.gulanbar@jichi.ac.jp (G.A.); manabu.takamatsu@jfcr.or.jp (M.T.); kenji.nishida@jfcr.or.jp (K.N.); kentakeuchi-tky@umin.net (K.T.); 4 Division of Pathology, Cancer Institute Hospital, Japanese Foundation for Cancer Research (JFCR), 3-8-31 Ariake, Koto-ku, Tokyo 135-8550, Japan; 5 Division of Tumor Pathology, Jichi Medical University, 3311-1 Yakushiji, Shimotsuke 329-0431, Japan; amori.mahmut@jichi.ac.jp; 6 Department of Pathology, Saitama Cancer Center, 780 Komuro, Ina, Kita-adachi-gun, Saitama 362-0806, Japan; hkanda@saitama-pho.jp; 7 Division of Hepatobiliary and Pancreatic Surgery, Cancer Institute Hospital, Japanese Foundation for Cancer Research (JFCR), 3-8-31 Ariake, Koto-ku, Tokyo 135-8550, Japan; yu.takahashi@jfcr.or.jp; 8 Pathology Project for Molecular Targets, Cancer Institute, Japanese Foundation for Cancer Research (JFCR), 3-8-31 Ariake, Koto-ku, Tokyo 135-8550, Japan

**Keywords:** BFT, enterotoxigenic *Bacteroides fragilis*, colorectal carcinoma, liver metastasis, tumor microbiome, tumor microenvironment

## Abstract

**Background**: Enterotoxigenic *Bacteroides fragilis* (ETBF) carries the *bft* toxin gene, which influences the host immune response and inflammatory pathways and promotes colorectal cancer (CRC). This study investigated the potential role of ETBF in CRC liver metastasis. **Methods**: We reviewed the records of 226 consecutive patients who underwent curative-intent (R0) resection of CRC liver metastases. ETBF DNA in fresh-frozen metastasis specimens was quantified using droplet digital PCR (ddPCR). Patients were grouped into very-low (≤80%; *N* = 178), low (80–90%; *N* = 24), and high (>90%; *N* = 24) ETBF-DNA groups. Three tissue cores per specimen were stained for CD8, CD4, CD20, FOXP3, CD68, and CD163, and immune-cell densities were measured digitally (cells/mm^2^). **Results**: ETBF DNA was detected in 219 of 226 lesions (96.9%). The densities of cytotoxic CD8^+^ T-cells, effector CD4^+^ T-cells, CD20^+^ B-cells, and CD163^+^ macrophages did not differ significantly by ETBF-DNA group (*P*_trend_ all > 0.12). FOXP3^+^ regulatory T-cells (Tregs) decreased (*P*_trend_ = 0.010), and CD68^+^ macrophages increased (*P*_trend_ = 0.020) as ETBF-DNA levels increased. ETBF-DNA levels in CRC liver metastases were not associated with disease-free survival or overall survival or serum C-reactive protein levels. **Conclusions**: ETBF was present in almost all CRC liver metastases. Higher ETBF levels were associated with a tumor-immune microenvironment enriched in CD68^+^ macrophages and deficient in FOXP3^+^ Tregs, suggesting that ETBF facilitates immune evasion without loss of effector lymphocytes. Although ETBF-DNA levels did not predict survival in this single-center cohort, the potential role of ETBF in immune remodeling and as a candidate biomarker and therapeutic target in metastatic CRC warrants further study.

## Simple Summary

Bacteria living inside tumors can shape how cancers grow. We examined one such microbe, enterotoxigenic *Bacteroides fragilis* (ETBF), in liver metastases removed from 226 patients with colorectal cancer. A highly sensitive DNA test detected ETBF in almost every tumor. Metastases carrying more bacterial DNA contained a greater number of macrophages and fewer regulatory T cells indicating an immune imbalance that can allow cancer to escape the body’s defenses, whereas the numbers of cytotoxic T cells and B cells were not affected by the amount of EBTF DNA in liver metastases. Although the bacterial load did not predict how long patients survived, its association with these immune shifts highlights a possible hidden role of ETBF in metastatic colorectal cancer. These results raise the possibility of diagnosing or treating metastatic colorectal cancer by targeting the bacterium or the immune changes it triggers.

## 1. Introduction

Colorectal cancer (CRC) is one of the most common malignancies worldwide and a leading cause of cancer-related death [1,2]. Approximately 35–55% of patients diagnosed with CRC eventually develop liver metastases [3], which account for more than 60% of CRC-related deaths [4,5]. Surgical resection remains the primary curative approach for liver metastases; however, the recurrence rate after resection ranges from 50% to 85% [6,7,8,9,10]. The 5-year survival rate for patients with unresectable liver metastases is less than 5%; therefore, further research is needed to elucidate factors contributing to CRC metastatic progression, particularly those within the hepatic microenvironment [11,12,13].

Advancements in metagenomic analysis have demonstrated that intratumoral bacteria play a critical role in modulating the immune response in multiple tumor types [14,15,16]. Among these microbes, enterotoxigenic *Bacteroides fragilis* (ETBF) plays a role in tumor progression [17]. ETBF carries the *bft* gene, which encodes *Bacteroides fragilis* toxin (BFT), which promotes CRC tumorigenesis by influencing the host immune response and inflammatory pathways [18]. BFT activates key signaling cascades, including the Wnt/β-catenin and NF-κB pathways [19,20]. Additionally, ETBF is associated with the high-CpG island-methylator phenotype and *BRAF* mutations in CRC, suggesting that it may influence tumor molecular characteristics [21]. Previous studies have established that ETBF plays a role in CRC initiation and progression; however, its effects have been investigated predominantly within the intestinal tract, and its potential role in CRC liver metastases has not been investigated previously.

Recent studies suggest that gut microbiota can influence distant metastases at sites beyond the intestinal environment, with intratumoral bacteria possibly reaching metastatic lesions by hematogenous dissemination [22]. Our previous research has demonstrated that *Fusobacterium nucleatum* and *pks*-carrying *Escherichia coli* may play a role in CRC liver metastases and tumor progression [23,24].

In this study, we assessed the presence of ETBF in CRC liver metastases and explored the association between the amount of ETBF-DNA present and the systemic and local immune responses as well as metastases in other organs. We quantified ETBF DNA in fresh-frozen specimens of CRC liver metastases and evaluated the tumor-immune microenvironment (TIME) using formalin-fixed paraffin-embedded (FFPE) specimens. Additionally, we analyzed clinical data, including serum C-reactive protein (CRP) levels and the number of other organs with metastases following R0 metastasectomy to elucidate the potential role of ETBF in the dynamics of CRC liver metastasis.

## 2. Materials and Methods

### 2.1. Patients and Specimens

This study included surgical specimens from patients diagnosed with CRC who underwent R0 hepatic metastasectomy at the Cancer Institute Hospital of the Japanese Foundation for Cancer Research (JFCR), Tokyo, Japan, between January 2005 and December 2015. Consecutive cases were included, and specimens were collected from metastatic liver lesions. The resected tissues were divided and preserved as fresh-frozen specimens and FFPE blocks. Fresh specimens were rapidly frozen in liquid nitrogen within 20 min of excision. The inclusion criteria were as follows: (1) patients who underwent surgical resection of colorectal adenocarcinoma at the Cancer Institute Hospital of JFCR and subsequently developed liver metastasis for which a R0 metastasectomy was performed; or (2) patients who were referred to the Cancer Institute Hospital of JFCR with colorectal adenocarcinoma liver metastasis and underwent R0 metastasectomy at the institution. Patients were only included if they had an adequate quantity of fresh-frozen and FFPE liver metastasis tissue samples. Patients who lacked sufficient tissue volume for analysis or whose clinical or pathological data were incomplete were excluded. Data on patient age, sex, preoperative laboratory findings, primary tumor site, number of hepatic metastases, prior chemotherapy history, and number of organs involved at recurrence were retrieved from electronic health records. Metastatic and recurrent involvement was evaluated in the brain, lungs, liver, adrenal glands, peritoneum, spleen, skeletal system, and lymph nodes. Because CRC liver metastases are frequently multiple and highly heterogeneous in size, the metastatic burden was quantified by multiplicity (single vs. multiple lesions and the total lesion count) rather than by the diameter or volume of individual lesions. All medical records were reviewed by members of the research team. All procedures in this study were performed in accordance with the ethical standards outlined in the Declaration of Helsinki and were approved by the Institutional Review Board of JFCR (approval number: 2016–1087, dated 27 September 2016). The requirement for informed consent was waived owing to the retrospective study design.

### 2.2. Pathological Analysis

The diagnosis of liver metastases was confirmed by two pathologists (Y.S. and K.I.) according to the fifth edition of the World Health Organization guidelines [25] using 4-μm-thick FFPE tissue sections stained with hematoxylin and eosin (H&E). The same H&E slides were subsequently reviewed to mark viable tumor areas while avoiding necrotic or hemorrhagic zones; this annotation guided the representative core sampling described in Section 2.2.1.

#### 2.2.1. Tissue Microarrays

Tissue microarrays (TMAs) were constructed using FFPE tumor specimens to enable immunohistochemical evaluation of tumors and tumor-infiltrating immune cells. Donor paraffin block regions were cored with a 2-mm needle and repositioned into a recipient block using a KIN-1 manual tissue arrayer (Azumaya, Tokyo, Japan). Three cores exemplifying the dominant histological characteristics were chosen from each metastatic tumor specimen. The resulting TMA sections, cut in 4-µm thick sections, were then employed for subsequent immunohistochemical analyses. Three representative 2-mm tissue cores were selected for each metastatic tumor, based on the H&E-guided annotations. This three-core strategy has been shown to have high concordance with whole-slide immune composition [26,27]. The resulting 4-µm-thick TMA sections were used for subsequent immunohistochemical analyses.

#### 2.2.2. Immunohistochemistry

Immunohistochemical staining was performed to evaluate tumor and immune cells within the TIME. The mismatch-repair (MMR) proteins MLH1 (1:100; clone ES05; Leica Biosystems, Newcastle, UK), MSH2 (1:500; clone G219-1129; BD Biosciences, Franklin Lakes, NJ, USA), MSH6 (1:500; clone EPR3945; Abcam, Cambridge, MA, USA), and PMS2 (1:100; clone A16-4; BD Biosciences) were evaluated. Immune-cell infiltration was then assessed using mouse monoclonal antibodies against CD8 (1:3; clone C8/144B; cat. 413201; Nichirei, Tokyo, Japan), CD4 (1:2; clone 4B12; cat. 413951; Nichirei), CD20 (1:800; clone L26; cat. NCL-L-CD20-L26; Leica Biosystems), FOXP3 (1:100; clone 236A/E7; cat. ab20034; Abcam), CD163 (1:1200; clone 10D6; cat. CD163-L-CE; Leica Biosystems), and CD68 (1:1000; clone KP-1; cat. M0814; Dako, Glostrup, Denmark). To unmask CD68 antigens, sections were incubated with protease K (1:200) for 5 min, whereas all the other epitopes underwent heat-induced retrieval in EDTA-surfactant buffer (pH 9.0) at 100 °C for 20 min. Adjacent nonneoplastic liver tissue, including hepatocytes, bile ducts, lymphocytes, and nerves, from the same TMA block served as positive and negative controls. Staining was carried out on a Bond-III automated platform (Leica Microsystems, Wetzlar, Germany), with visualization achieved via the Bond Polymer Refine Detection Kit (Leica Microsystems).

#### 2.2.3. Immunohistochemical Evaluation of Tissue Microarrays

We evaluated tumor-infiltrating immune cells and MMR protein expression in the TMAs. MLH1, PMS2, MSH2, and MSH6 were examined as indicators of MMR protein function, and tumors were considered to have lost MMR protein expression if nuclear staining was absent in tumor cells but present in lymphocytes. Three TMA cores of each tumor specimen were assessed for each MMR protein, and tumors were classified as having lost protein expression if all three cores lacked expression. Tumors lacking any of the four MMR proteins were defined as having deficient MMR (dMMR). All sections were evaluated independently by two pathologists who were blind to the case details, and ambiguous sections were reviewed by a third pathologist. Digital analysis of immunostained sections was conducted using the NanoZoomer Digital Pathology System (Hamamatsu Photonics KK, Shizuoka, Japan) at ×40 magnification with a resolution of 0.55 pixel/μm. The images were converted into the proprietary NanoZoomer format and analyzed using Fiji (version 2.14.0), an open-source biological image analysis software [28]. Representative microscopic fields from scanned TMA images were selected, and the number of tumor-infiltrating immune cells was quantified using Fiji. For each tumor, the mean number of immune cells in three tissue cores was calculated as a measure of immune-cell infiltration.

### 2.3. DNA Extraction and Quantitative PCR for ETBF

DNA was extracted from fresh-frozen CRC liver metastasis tissue specimens using the NucleoSpin Tissue Kit (Takara Bio Inc., Otsu, Japan) and quantified using a Nanodrop ND-1000 spectrophotometer (Thermo Fisher Scientific Inc., Waltham, MA, USA). The initial PCR was conducted using 25 μL of sample solution comprising 100 ng of extracted DNA and specific primers designed to detect ETBF-specific sequences [29] (Table S1) according to the conditions shown in Table S2. Regarding primer specificity, we used primer sequences targeting the *bft* gene that have been widely used and validated in previous studies [29]. For quantification purposes, the initial PCR product obtained from ETBF was diluted 107-fold and EvaGreen-based droplet digital PCR (ddPCR) with 1 μL of the diluted sample was utilized subsequently. ETBF copy numbers were normalized to human genomic DNA using primers for the SLCO2A1 reference gene (Table S1) [30]. Each ddPCR reaction contained 10 ng of genomic DNA and was run on a QX200 Droplet Digital PCR System (Bio-Rad, Hercules, CA, USA); data were processed with QuantaSoft v1.7.4.0917. To monitor contamination, a no-template control (NTC) without DNA was included in every first-round PCR, and its product was carried forward at a 1 × 10^−7^ dilution into the ddPCR. In addition, ddPCR-only blanks containing reagents, but no template or pre-amplified product were added to each ddPCR plate. No ETBF signal was detected in any of the negative controls, and laboratory contamination was therefore considered negligible. Adjacent normal liver tissue was not used as a negative control because such tissue may harbor low-level microbiota and therefore cannot serve as a true blank. The contamination assessment relied exclusively on the technical blanks described above [31]. Stringent contamination control measures recommended for low-biomass microbiome studies were observed throughout to ensure accurate and reliable DNA quantification [32]. Because ddPCR is highly sensitive, careful evaluation of background signals was required. A representative scatter plot (Figure S1) revealed that in the NTC carried through the two-step protocol (Lane 2) a small droplet cluster appeared at approximately 5000–9000 arbitrary units fluorescence; agarose-gel electrophoresis confirmed by size verification that these droplets were primer-dimers rather than true amplicons, and they were therefore classified as negative. In contrast, positive droplets in the positive control (Lane 1) formed a clearly separated high-amplitude population (>15,000 arbitrary units). The lower edge of this positive cluster was used as the positivity threshold and applied uniformly to all samples, yielding a binary classification of droplets as either positive or negative.

### 2.4. Statistical Analysis

Statistical analyses were conducted using R software version 4.2.3 (The R Foundation for Statistical Computing, Vienna, Austria). Patients were categorized into high, low, and very-low ETBF-DNA groups according to the ETBF-DNA level in the liver metastasis. Continuous variables were compared between the three groups using one-way ANOVA or the Kruskal–Wallis test. The association between the number of metastatic sites and ETBF-DNA group was assessed using the chi-squared test, and the trend between ETBF-DNA level (as an ordinal variable) and the density of tumor-infiltrating immune cells and number of metastases were assessed using the Jonckheere–Terpstra test. Survival outcomes, including disease-free survival (DFS) and overall survival (OS), were estimated using Kaplan–Meier curves and compared using log-rank tests. DFS was defined as the time from curative-intent hepatic resection to the first documented recurrence or death from any cause, whichever occurred first. OS was defined as the time from hepatic resection to death from any cause; patients alive at last follow-up were censored on that date. The median follow-up was estimated using the reverse Kaplan–Meier method. Two-sided *p* values < 0.05 were considered statistically significant.

## 3. Results

### 3.1. Detection of ETBF in CRC Liver Metastasis Tissues

A total of 226 patients who had complete pathological records and an adequate quantity of fresh-frozen and FFPE liver metastasis specimens were included. ETBF DNA was found in 219 (96.9%) of the 226 CRC liver metastasis specimens examined. The amount of ETBF D A detected was classified as very low (≤80%) in 178 patients, low (80–90%) in 24 patients, and high (>90%) in 24 patients. The ETBF displayed two elbows at 0.08 and 0.16. These points delimited the lower 80% of essentially zero values, a transitional band (0.08–0.16), and a steep rise in the top decile (>0.16) (Figure S2A). We, therefore, defined very-low (≤0.08), low (0.08–0.16), and high (>0.16) strata. To avoid arbitrary quartile-based cutoffs, we used statistical inflection points in the empirical distribution of ETBF (Figure S2B). Patient age, sex, number of liver metastases, chemotherapy, timing of metastasis, tumor differentiation, MMR protein status, and serum CRP levels did not differ significantly by ETBF-DNA group (Table 1).

### 3.2. ETBF and the TIME in CRC Liver Metastasis Tissues

**Figure 1:**
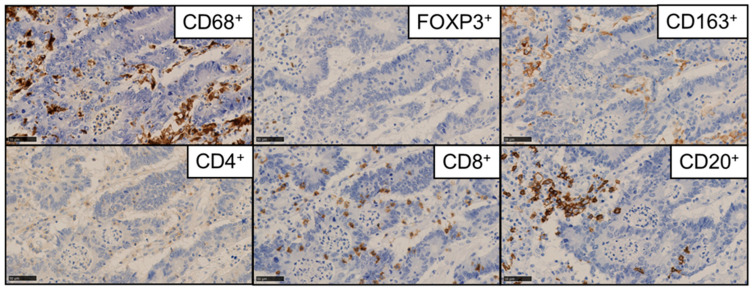
Immunohistochemistry of CD8^+^, CD4^+^, CD20^+^, FOXP3^+^, CD163^+^, and CD68^+^ cells in colorectal cancer liver metastasis tissues. Scale bar = 50 μm.

In the TMA cores, tumor-infiltrating immune cells were relatively uniform in distribution, appearing in dispersed or clustered formations. The quantified densities of CD8^+^, CD4^+^, CD20^+^, FOXP3^+^, CD163^+^, and CD68^+^ cells are shown in Figure 1.

**Figure 2:**
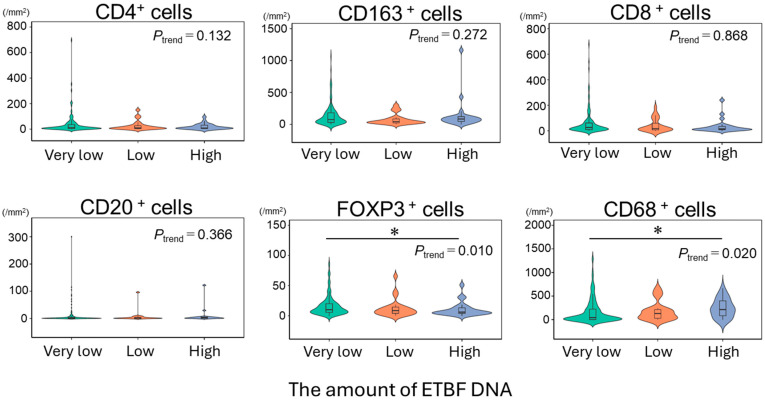
Violin plots showing the density of different types of immune cells according to the ETBF-DNA level. Violin plots display the densities of six immune-cell populations (CD4^+^, CD8^+^, CD20^+^, FOXP3^+^, CD163^+^, and CD68^+^) in three ETBF-DNA categories (very low, low, and high). The *X*-axis indicates the ETBF-DNA level in colorectal cancer liver metastases, and the *Y*-axis shows the corresponding immune-cell density. The Jonckheere–Terpstra test was used to determine *p* values for trend between ETBF-DNA levels (as an ordinal variable) and the density of tumor-infiltrating immune cells. * indicates *p* < 0.05. ETBF, enterotoxigenic *Bacteroides fragilis*.

The density of FOXP3^+^ regulatory T-cells (Tregs) decreased significantly as the ETBF-DNA level increased (*P*_trend_ = 0.010), with a 35% decrease between the very-low and high ETBF-DNA groups, whereas the density of CD68^+^ cells increased significantly as ETBF-DNA levels increased (*P*_trend_ = 0.020), with a 47% increase between the very-low and high ETBF-DNA groups. The densities of CD8^+^, CD4^+^, CD20^+^, and CD163^+^ cells did not differ significantly between ETBF-DNA groups (Table 2, Figure 2).

Log_2_ transformation provided similar results: The densities of FOXP3^+^ and CD68^+^ cells decreased and increased significantly, respectively, as the ETBF-DNA level increased (*p* = 0.012 and *p* = 0.008, respectively), whereas the density of the other cell types did not differ significantly between groups (Figure S3). To assess the robustness of our findings to cut off selection, we performed an additional binary stratification (Low vs. High), using the median as the cut-point. This binary stratification revealed a non-significant decrease in the density of FOXP3^+^ cells (15.98 ± 16.60 vs. 13.73 ± 15.23 cells/mm^2^; *p* = 0.294), and a significant increase in the density of CD68^+^ cells (mean ± SD: 138.08 ± 196.93 vs. 216.50 ± 275.64 cells/mm^2^; *p* = 0.015) as the ETBF-DNA level increased, indicating that our findings were not sensitive to the choice of cut-points (Table S3).

### 3.3. Diversity of Metastatic Organs Involved in Recurrence

A total of 147 patients had a recurrence of extracolonic metastasis following R0 resection with hepatic metastasectomy, of whom 116, 15, and 16 were in the very-low, low, and high ETBF-DNA groups, respectively. The number of metastatic organs involved in recurrence did not differ significantly according to the ETBF-DNA group (Table S4).

### 3.4. ETBF in CRC Liver Metastasis Tissues and Survival

**Figure 3:**
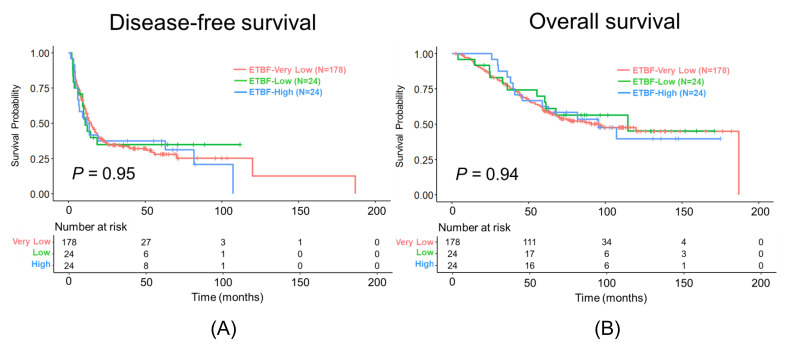
Kaplan–Meier curves of (**A**) disease-free survival and (**B**) overall survival after curative-intent resection. Blue, green, and red lines represent the survival probability in high, low, and very-low ETBF-DNA groups, respectively. ETBF, enterotoxigenic *Bacteroides fragilis*.

DFS and OS did not differ significantly according to the ETBF-DNA group (log-rank *p* = 0.95 and 0.94, respectively; Figure 3). The median length of follow-up, estimated using the reverse Kaplan–Meier estimate, was 95.8 (95% CI: 89.3–104.1) months for OS. The number of events was 178, 24, and 24 for the Very Low, Low, and High groups, respectively. The median DFS and OS are provided in Table S5. Kaplan–Meier curves for DFS and OS stratified by ETBF load and chemotherapy status, are presented in Figures S4 and S5, respectively.

## 4. Discussion

ETBF DNA was detected by ddPCR in 97% of colorectal-liver metastases, which is considerably higher than the prevalence detected in previous studies using conventional assays. This enabled us to study the association between bacterial load and the local TIME. Lesions in the high ETBF-DNA group showed a pronounced enrichment of CD68^+^ macrophages and a depletion of FOXP3^+^ Tregs, whereas CD8^+^ and CD4^+^ T-cell counts did not differ significantly according to the ETBF-DNA level. This pattern suggests that ETBF does not indiscriminately suppress lymphocyte infiltration but rather re-shapes the myeloid and regulatory compartments, leading to a macrophage-rich, Treg-poor TIME. Given that tumor-associated macrophages can promote angiogenesis and immune suppression, whereas Tregs suppress inflammation, such a shift is likely to create conditions that favor metastatic growth without a decrease in the cytotoxic T-cell count.

In our cohort, DFS and OS were not affected by the presence of ETBF-DNA or the ETBF-DNA level, consistent with a previous study that found no association between ETBF status and survival in patients with primary CRC [21]. The ETBF-DNA level was also not associated with the serum CRP level or the number of metastatic organs involved in recurrence, suggesting that its influence may be confined to local immune remodeling rather than to overt clinical aggressiveness. However, detailed profiling of intratumoral lymphoid and myeloid subsets, including CD4^+^ and CD8^+^ T-cell, FOXP3^+^ Treg, CD20^+^ B-cell, and CD68^+^ or CD163^+^ macrophage counts, are crucial for predicting prognosis, as composite indices such as the metastasis immunoscore and the macrophage immunoscore are more accurate than single-marker analyses at predicting prognosis [33,34,35,36]. Future studies should assess whether incorporating ETBF quantification into such multi-parameter immune signatures can refine risk stratification in metastatic CRC.

Although no statistically significant differences in DFS or OS were observed according to the ETBF DNA group, the ETBF-associated increase in CD68^+^ macrophages and decrease in FOXP3^+^ Tregs might modulate finer clinical behaviors such as treatment responsiveness and metastatic tropism. Tumor-associated macrophages have been reported to confer resistance to 5-fluorouracil [37], and Treg-low TMEs appear more responsive to immune checkpoint blockade [38], and macrophage-rich TMEs might facilitate vessel co-option, which is linked to liver-dominant spread and resistance to anti-VEGF therapy [39]. Therefore, prospective studies that integrate ETBF quantification with treatment-response data and organ-specific recurrence patterns are warranted.

ETBF appears to skew the TIME toward a microenvironment that favors metastatic growth and immune escape in CRC liver metastatic lesions. ETBF releases BFT, prompting tumor and stromal cells to secrete interleukin (IL)-6, IL-1β, and IL-23 [18,40]. These cytokines draw circulating monocytes into the metastatic niche, where they mature into CD68^+^ macrophages that support angiogenesis and suppress cytotoxic T-cell activity [41]. IL-6 simultaneously prevents naïve CD4^+^ cells away from differentiating into FOXP3^+^ Tregs and an inflammatory Th17 profile [42]. Because Tregs normally suppress inflammation, Treg depletion coupled with an influx of tumor-promoting macrophages, favors pro-tumor signals. The ETBF-associated increase in CD68^+^ macrophages, together with the decrease in FOXP3^+^ T-regs, suggests that ETBF may remodel the TIME through two complementary routes. In addition to cytokine-mediated effects, *Bacteroides fragilis* toxin (bft) has been shown to activate the NF-κB and MAPK signaling pathways in tumor and stromal cells [43,44]. This may amplify inflammatory responses and modulate immune-cell recruitment. IL-6 can also activate the STAT3 signaling pathway in macrophages and T cells. This pathway has been linked to M2 polarization and inhibition of Treg differentiation [45,46]. These pathways provide a plausible mechanistic link between ETBF exposure and the observed TIME shift. Moreover, direct host–microbe interactions might influence the TIME shift. Pattern-recognition receptors such as TLR2 or NOD1 expressed on innate immune cells such as macrophages or dendritic cells may detect ETBF-derived molecules and contribute to shaping the local TIME [47,48,49]. Further functional studies, including in vitro co-culture assays and in vivo loss-of-function models, are warranted to clarify these possibilities. This dual shift helps explain why higher ETBF-DNA loads in our study were associated with fewer FOXP3^+^ cells and more CD68^+^ macrophages, creating a TIME that favored metastatic growth and immune escape.

Detecting bacterial DNA in tumor tissue is technically demanding because the copy number is low, DNA fragments readily in FFPE blocks, and conventional quantitative PCR (qPCR) often cannot differentiate between true signal and background noise. Consequently, previous studies have reported detection rates of only 4% for *F. nucleatum* in CRC liver metastases [50], and 37% for *pks*-carrying *E. coli* [51], and 9% for ETBF in primary CRC tumors [21]. Although ddPCR has improved the yield for *F. nucleatum* [52], to our knowledge, this is the first study to use ddPCR to detect ETBF DNA in metastatic CRC lesions. By analyzing fresh-frozen specimens, thereby avoiding formalin-induced DNA damage and introducing a two-step template-enrichment protocol before ddPCR quantification [23,24], we increased the ETBF detection rate to 97%. This improvement over standard qPCR not only confirms that ETBF is present in almost all CRC liver metastases but also provides a reliable method of assessing relationships between the ETBF-DNA level and immune-cell populations. These results suggest that ETBF may play a potentially important role in the biology of metastatic CRC that has not previously been recognized.

This study has several limitations. First, its retrospective design precludes causal inference and is susceptible to bias. Second, ETBF was quantified in the single, largest-diameter metastasis in each patient; therefore, intertumoral heterogeneity may have been missed. Third, immunophenotyping relied on TMAs, which capture only small tumor regions. Despite analyzing three cores per case, some spatial variation in the TIME is likely to have been missed. Fourth, the statistical power to detected differences according to the ETBF-DNA level was limited owing to the limited sample size. Fifth, technical constraints prevented direct visualization of ETBF by fluorescence in situ hybridization (FISH) or similar methods, so the precise spatial relationship between ETBF and immune cells remains unknown. Sixth, we measured ETBF-DNA copy number but not *bft* transcriptional activity, so we were unable to assess whether ETBF was metabolically active in metastases. Seventh, the immune profiling panel focused on T-cell, B-cell, and macrophage lineages and did not include neutrophils, dendritic cells, or myeloid-derived suppressor cells. Eighth, the heterogeneity in immune-cell populations within the very-low ETBF-DNA group suggests complexity of interactions between the microbiota, tumor cells, and host immune responses in shaping the TIME. This warrants further research. Ninth, because the present study is correlative, additional functional work, including broad in vitro macrophage-polarization, cytokine-release assays, and pilot co-culture or animal models, are needed to determine whether bft-driven signaling can reproduce the TIME shift observed in this study. Finally, all patients were treated in a single Japanese center, so the results may not be generalizable to patients with metastatic CRC in other settings. Therefore, multicenter, prospective studies in ethnically and geographically diverse cohorts are needed to confirm the generalizability of these findings and the clinical utility of ETBF profiling in metastatic CRC.

## 5. Conclusions

By pairing fresh-frozen tissue with an optimized ddPCR workflow, we detected ETBF DNA in almost all CRC liver metastases and quantified its load with high precision. Higher ETBF-DNA levels were associated with lower FOXP3^+^ Treg and higher CD68^+^ macrophage counts, suggesting that ETBF influences the metastatic TIME. Although the ETBF level did not affect survival in our cohort, the clear immune shift points to ETBF as a possible biomarker and therapeutic target in metastatic CRC. Prospective, multicenter studies, ideally incorporating spatial profiling and functional assays, are required to confirm these associations and to test whether modulating ETBF or its downstream cytokine pathways improves patient outcomes.

## Figures and Tables

**Table 1:** Clinicopathological features according to the amount of ETBF in CRC liver metastasis tissues.

Variable	Total (*N* = 226)	ETBF-DNA Level in CRC Liver Metastatic Tissues			*p* Value
		Very Low (*N* = 178)	Low (*N* = 24)	High (*N* = 24)	
Age (years) ^†^	62.3 (11.0)	62.9 (11.0)	60.5 (11.5)	59.3 (10.3)	0.190
Sex ^§^					0.777
Female	144 (63.7)	112 (62.9)	15 (62.5)	17 (70.8)	
Male	82 (36.3)	66 (37.1)	9 (37.5)	7 (29.2)	
Serum CRP (mg/dL) ^†^	0.4 (1.0)	0.3 (0.8)	0.6 (1.2)	0.7 (1.9)	0.226
Number of liver metastasis ^‡^	2 (1–5)	2 (1–6)	1 (1–2.25)	2 (1–4)	0.082
Primary tumor location ^§^					0.556
Right side	47 (20.9)	40 (22.5)	3 (12.5)	4 (17.4)	
Left side	178 (79.1)	138 (77.5)	21 (87.5)	19 (82.6)	
Preoperative chemotherapy ^§^					0.295
None	130 (55.3)	108 (57.8)	10 (41.7)	12 (50.0)	
received	105 (44.7)	79 (42.2)	14 (58.3)	12 (50.0)	
Adjuvant chemotherapy ^§^					0.840
None	124 (52.8)	98 (52.4)	14 (58.3)	12 (50.0)	
received	111(47.2)	89 (47.6)	10 (41.7)	12 (50.0)	
Timing of metastasis ^§^					0.293
Synchronous	122 (54.0)	101 (56.7)	10 (41.7)	11 (45.8)	
Metachronous	104 (46.0)	77 (43.3)	14 (58.3)	13 (54.2)	
Tumor differentiation ^§^					0.850
Well to moderate	214 (95.1)	167 (94.4)	23 (95.8)	24 (100)	
Poor	11 (4.9)	10 (5.6)	1 (4.2)	0 (0)	
Mismatch repair protein ^§^					0.167
Intact	219 (96.9)	174 (97.8)	23 (95.8)	22 (91.7)	
Deficient	7 (3.1)	4 (2.2)	1 (4.2)	2 (8.3)	

CRC, colorectal cancer; CRP, C-reactive protein; ETBF, enterotoxigenic *Bacteroides* *fragilis*. ^§^ Data presented as *n* (%); ^†^ Data presented as mean (± standard deviation); ^‡^ Data presented as median (interquartile range).

**Table 2:** ETBF and density of immune cells in CRC liver metastasis tissues.

Cell Type	ETBF-DNA Level in CRC Liver Metastasis			*p* Value for Trend
	Very Low Mean ± SD (cells/mm^2^) (*N* = 178)	Low Mean ± SD (cells/mm^2^) (*N* = 24)	High Mean ± SD (cells/mm^2^) (*N* = 24)	
CD8^+^ cells	57.0 (±94.5)	41.5 (±51.9)	34.0 (±53.4)	0.868
CD4^+^ cells	33.4 (±72.0)	26.1 (±37.9)	18.8 (±22.7)	0.132
CD20^+^ cells	7.9 (±27.9)	6.5 (±19.3)	8.6 (±24.9)	0.366
FOXP3^+^ cells	16.0 (±16.5)	12.2 (±15.0)	10.4 (±11.8)	0.016
CD163^+^ cells	133.8 (±165.9)	77.0 (±85.4)	135.7 (±235.1)	0.272
CD68^+^ cells	167.3 (±251.7)	179.3 (±200.7)	245.9 (±197.2)	0.020

CRC, colorectal cancer; ETBF, enterotoxigenic *Bacteroides fragilis*. The Jonckheere–Terpstra test was used to determine *p* values for trend according to the ETBF-DNA level (as an ordinal variable) and the density of tumor-infiltrating immune cells.

## Data Availability

The datasets used and/or analyzed during the current study are available from the corresponding author on reasonable request.

## Supplementary Materials

The following supporting information can be downloaded at https://www.mdpi.com/article/10.3390/cancers17172733/s1: Table S1. Primer sequences of ETBF and *SLCO2A1*. Table S2. PCR conditions of ETBF and *SLCO2A1*. Table S3. Immune cell marker densities (mean ± SD) in low vs. high ETBF groups. Table S4. Number of organs with metastases according to the ETBF-DNA level in CRC liver metastasis tissues. Table S5. Survival outcomes by ETBF level. Figure S1. Representative ddPCR scatter plot illustrating positive and negative controls and sample classification. Figure S2. Empirical distribution of ETBF-DNA values used for threshold determination. Figure S3. Log2-transformed densities of tumor-infiltrating immune cells according to ETBF-DNA levels in CRC liver metastasis tissues. Figure S4. Disease-free survival stratified by ETBF category and chemotherapy status. Figure S5. Overall survival stratified by ETBF category and chemotherapy status.

## Abbreviations

The following abbreviations are used in this manuscript:

BFT	*Bacteroides fragilis* toxin
CRC	Colorectal cancer
CRP	C-reactive protein
ddPCR	Droplet digital PCR
DFS	Disease-free survival
dMMR	Deficient mismatch repair
ETBF	Enterotoxigenic *Bacteroides fragilis*
FFPE	Formalin-fixed paraffin-embedded
FISH	Fluorescence in situ hybridization
H&E	Hematoxylin and eosin
JFCR	Japanese Foundation for Cancer Research
MMR	Mismatch repair
NTC	No-template control
OS	Overall survival
SD	Standard deviation
Th17	T helper type 17
