# Supplementary material for: Association of Enterotoxigenic Bacteroides fragilis with Immune Modulation in Colorectal Cancer Liver Metastasis
Source: Cancers (Basel). 2025 Aug 22;17(17):2733. doi: 10.3390/cancers17172733 (PMC12427544; doi:10.3390/cancers17172733)
Supplement: Supplementary file 1 [file cancers-17-02733-s001.zip › cancers-3721080-supplementary.pdf]

Supplementary Information

Table S1. Primer sequences of ETBF and SLCO2A1.

Table S2. PCR conditions of ETBF and SLCO2A1.

Table S3. Immune cell marker densities (mean ± SD) in low vs. high ETBF groups.

Table S4. Number of organs with metastases according to the ETBF-DNA level present in CRC liver metastasis tissues.

Table S5. Survival outcomes by ETBF level.

Figure S1. Representative ddPCR scatter plot illustrating positive and negative controls, and sample classification.

Figure S2. Empirical distribution of ETBF-DNA values used for threshold determination.

Figure S3. Log2-transformed densities of tumor-infiltrating immune cells according to ETBF-DNA levels in CRC liver metastasis tissues.

Figure S4. Disease-free survival stratified by ETBF category and chemotherapy status.

Figure S5. Overall survival stratified by ETBF category and chemotherapy status.

Table S1. Primer sequences of ETBF and *SLCO2A1*

Target	Primer F (5′–3′)	Primer R (5′–3′)
ETBF	TGAAGTTAGTGCCCAGATGC	CAGTAAAGCCTTCCAGTCC
SLCO2A1	ATCCCCAAAGCACCTGGTTT	AGAGGCCAAGATAGTCCTGGTAA

ETBF, enterotoxigenic *Bacteroides fragilis.*

Table S2. PCR conditions of ETBF and *SLCO2A1*.

Target	Cycling Step	Temperature	Time	Cycles
ETBF	Initial denaturation	95°C	10 min	1
	Denaturation	95°C	10 s	40
	Annealing & extension	62°C	60 s	40
	Hold	4°C	∞ (hold)	1
SLCO2A1	Initial denaturation	95°C	10 min	1
	Denaturation	95°C	15 s	35
	Annealing & extension	60°C	60 s	35
	Hold	4°C	∞ (hold)	1

ETBF, enterotoxigenic *Bacteroides fragilis.*

Table S3. Immune cell marker densities (mean ± SD) in low vs. high ETBF groups.

Cell type	ETBF-DNA level in CRC liver metastasis		*P* value
	Low<br>Mean ± SD<br>(cells/mm^2)<br>(*N* = 113)	High<br>Mean ± SD<br>(cells/mm^2)<br>(*N* = 113)	
CD4+ cells	28.20 ± 47.26	33.89 ± 79.92	0.524
CD8+ cells	49.65 ± 80.46	58.46 ± 97.85	0.463
CD68+ cells	138.08 ± 196.93	216.50 ± 275.64	0.015
CD163+ cells	119.32 ± 151.08	136.22 ± 184.76	0.462
FOXP3+ cells	15.98 ± 16.60	13.73 ± 15.23	0.294
CD20+ cells	5.77 ± 15.97	9.81 ± 33.95	0.254

ETBF, enterotoxigenic *Bacteroides fragilis*; SD, standard deviation.

Table S4. Number of organs with metastases according to the ETBF-DNA level present in CRC liver metastasis tissues.

Number of organs involved	ETBF-DNA level			*P* value
	Very low<br>(*N* = 116)	Low<br>(*N* = 15)	High<br>(*N* = 16)	
1	90 (81.1%)	14 (93.3%)	15 (93.8%)	0.36
2	15 (13.5%)	0 (0%)	1 (6.2%)	
3	6 (5.4%)	0 (0%)	0 (0%)	
4	0 (0%)	0 (0%)	0 (0%)	
5	0 (0%)	1 (6.7%)	0 (0%)	

CRC, colorectal cancer; ETBF, enterotoxigenic *Bacteroides fragilis.*

Table S5. Survival outcomes by ETBF level.

Median (months)	Amount of ETBF DNA in CRC liver metastasis		
	Very low	Low	High
DFS	14.75 (11.93–18.73)	10.84 (9.17–Not reached)	12.71 (6.73–Not reached)
OS	95.86 (66.92–Not reached)	114.65 (60.48–Not reached)	95.53 (58.87–Not reached)

Data are presented as median (95% confidence interval). CRC, colorectal cancer; DFS, disease-free survival; ETBF, enterotoxigenic *Bacteroides fragilis*; OS, overall survival.

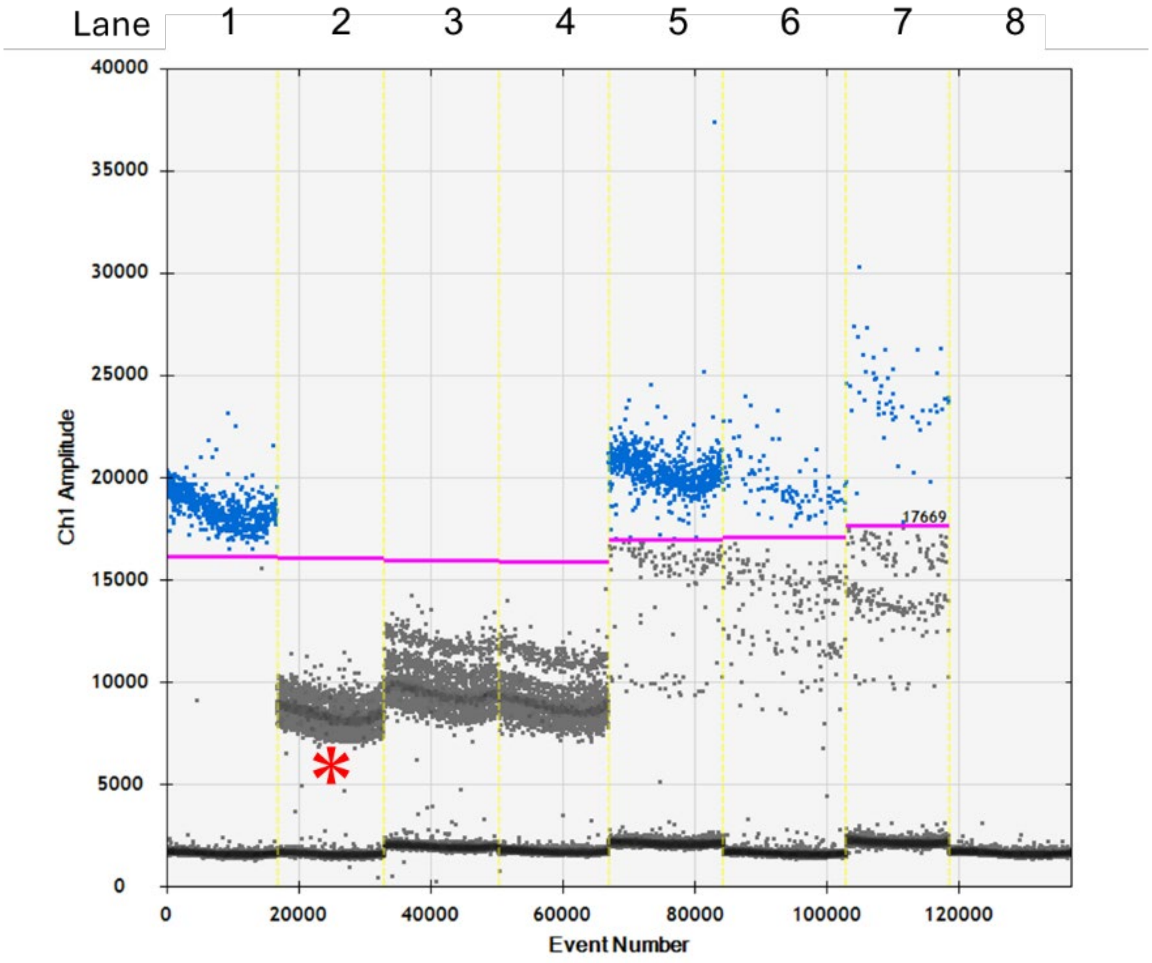

Figure S1. Representative ddPCR scatter plot illustrating positive and negative controls, and sample classification.

Droplet digital PCR (ddPCR) amplitude plot demonstrating the threshold (magenta line) utilized for determining positive events. Channel 1 (Ch1) amplitude is displayed on the Y-axis, with individual droplets represented by dots. Lane 1 indicates the positive control, while Lane 2 represents the negative control of the first PCR step. Lanes 3 and 4 depict samples classified as negative based on the established threshold. Lanes 5 to 7 illustrate samples determined to be positive. Lane 8 represents the ddPCR negative control.

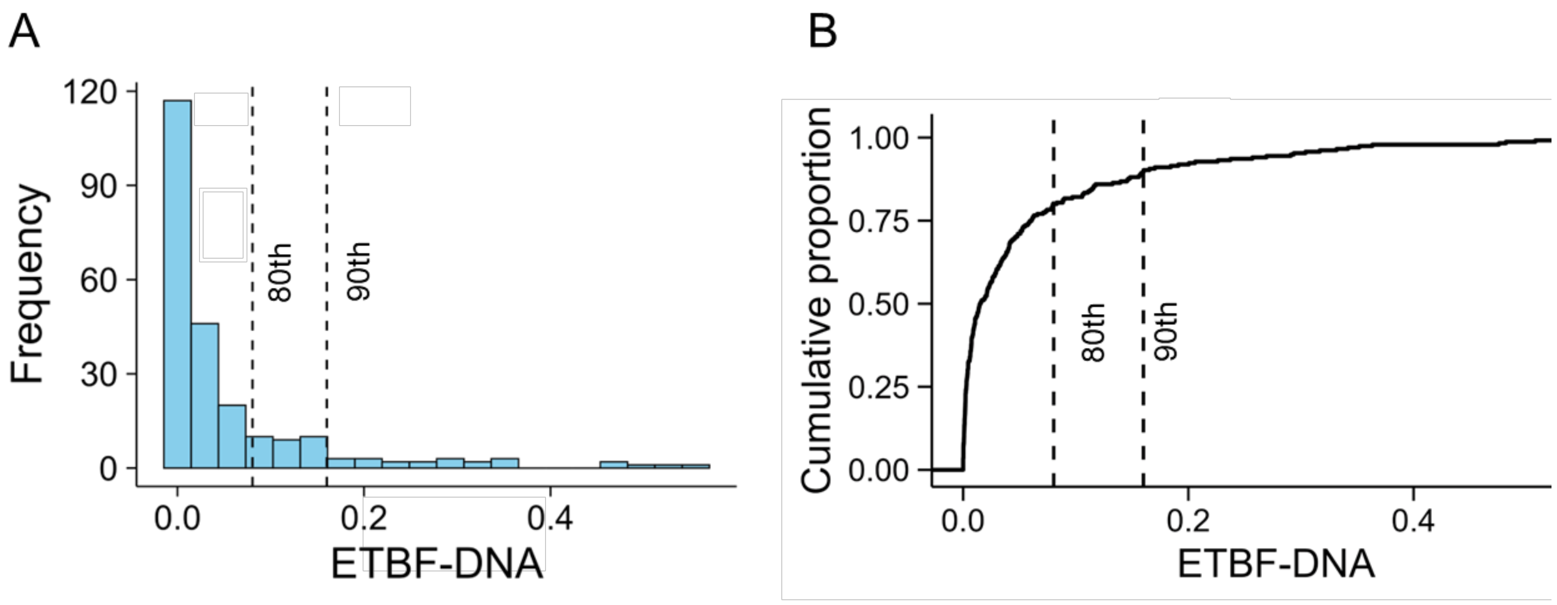

Figure S2. Empirical distribution of ETBF-DNA values used for threshold determination.

(A) Histogram of enterotoxigenic *Bacteroides fragilis* (ETBF)-DNA values with 80th and 90th percentiles marked by dashed lines. (B) Empirical cumulative distribution function (ECDF) highlighting inflection points at 0.08 and 0.16 used to define very low (≤0.08), low (0.08–0.16), and high (>0.16) ETBF-DNA levels.

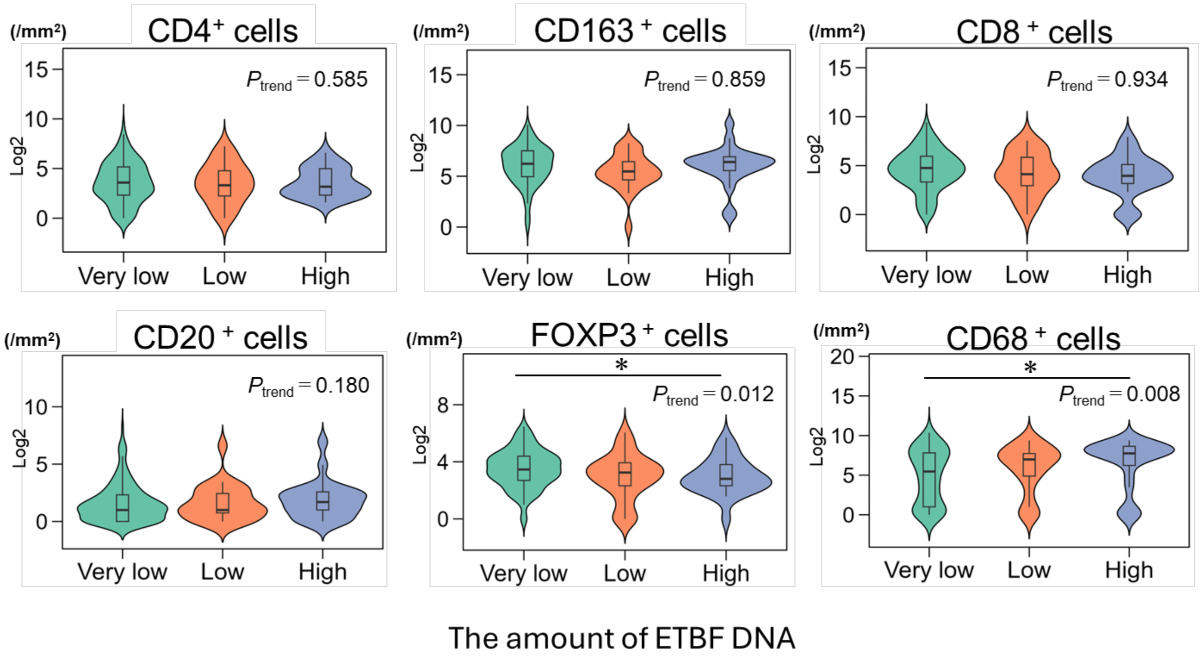

Figure S3. Log2-transformed densities of tumor-infiltrating immune cells according to ETBF-DNA levels in CRC liver metastasis tissues.

Violin plots showing the distribution of six types of tumor-infiltrating immune cells ($CD4^+$ cells, $CD163^+$ cells, $CD8^+$ cells, $CD20^+$ cells, $FOXP3^+$ cells, and $CD68^+$ cells) according to the amount of enterotoxigenic *Bacteroides fragilis* (ETBF) DNA in colorectal cancer liver metastasis tissues. The horizontal axis indicates the ordinal groups of ETBF-DNA levels (Very low, Low, High), and the vertical axis represents the log_2-transformed cell density (/mm²). *P* values for trend were calculated using the Jonckheere–Terpstra test. Asterisks (*) indicate statistical significance at $P < 0.05$.

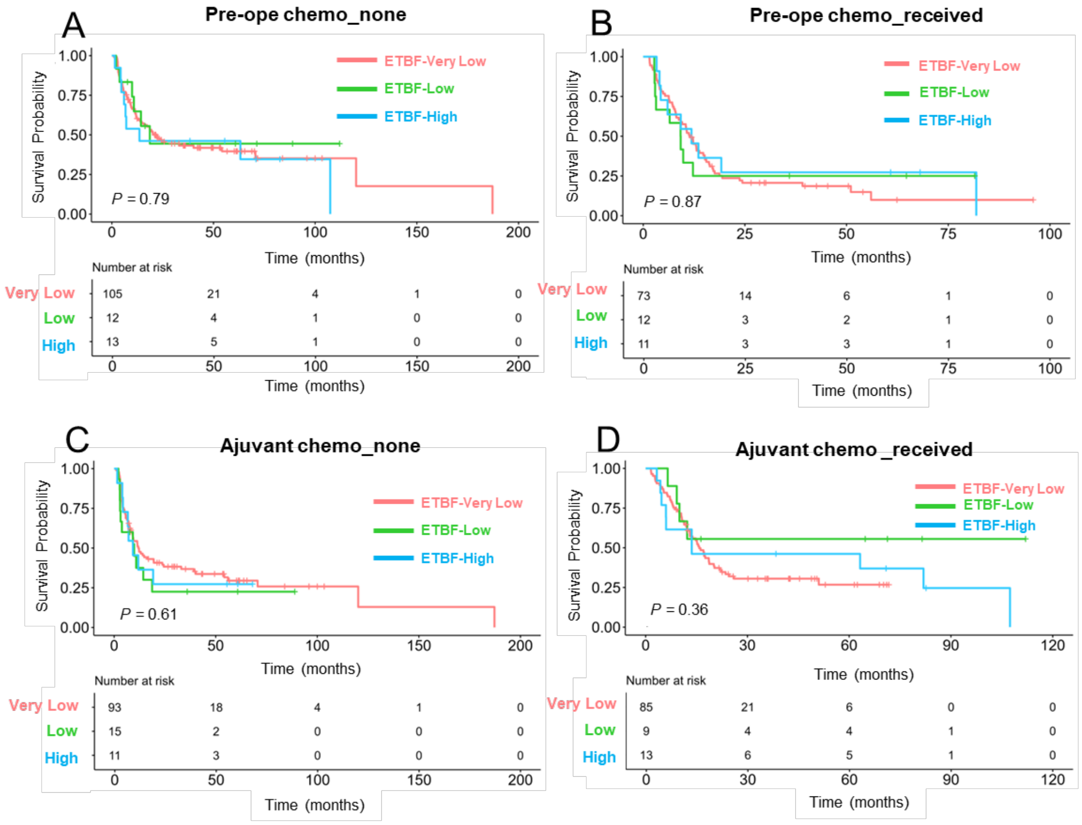

Figure S4. Disease-free survival stratified by ETBF category and chemotherapy status.

(A) Pre-operative chemotherapy none: Kaplan–Meier curve for disease-free survival in patients who received no pre-operative chemotherapy, stratified by enterotoxigenic *Bacteroides fragilis* (ETBF) abundance (very low, low, high). (B) Pre-operative chemotherapy received: Disease-free survival curve for patients who received pre-operative chemotherapy, stratified by ETBF abundance. (C) Adjuvant chemotherapy none: Disease-free survival curve for patients who underwent surgery without adjuvant chemotherapy, stratified by ETBF abundance. (D) Adjuvant chemotherapy received: Disease-free survival curve for patients who received adjuvant chemotherapy, stratified by ETBF status. *P* values are from log-rank tests. Risk tables beneath each panel indicate the number of patients at risk at the specified time points.

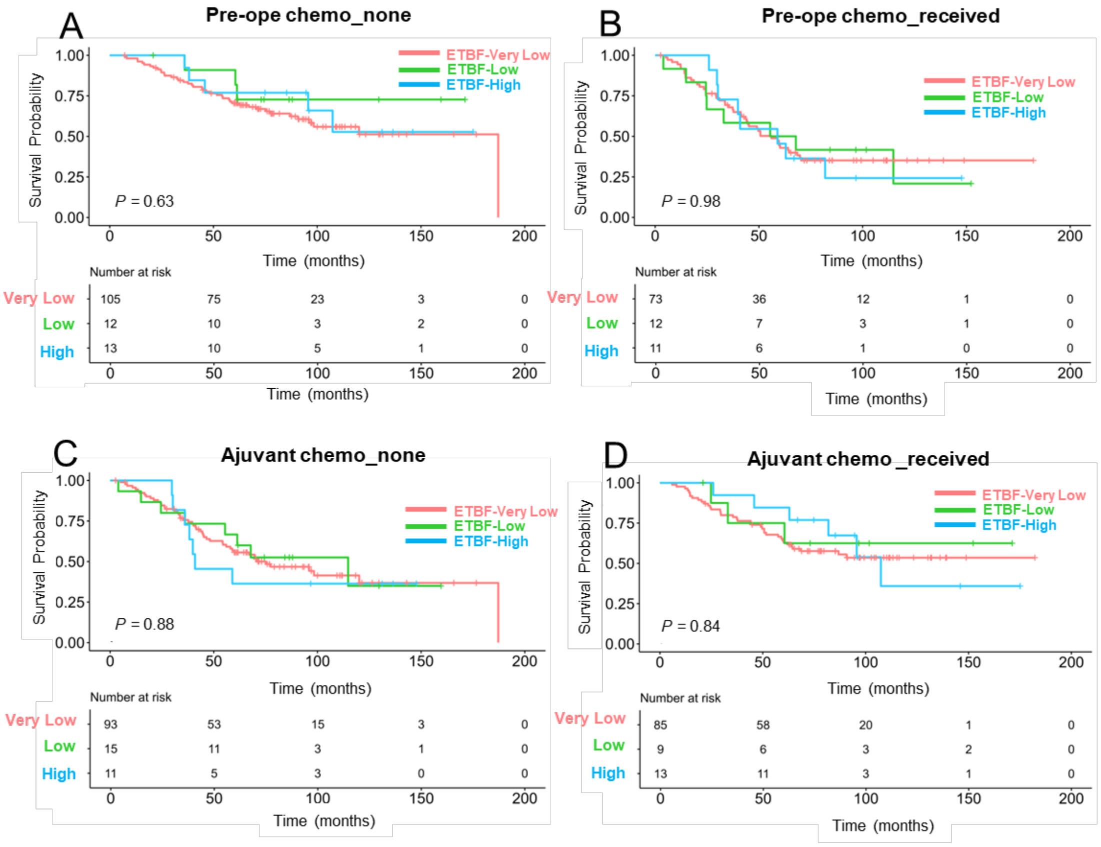

Figure S5. Overall survival stratified by ETBF category and chemotherapy status. (A) Pre-operative chemotherapy none: Kaplan–Meier curve for overall survival in patients who received no pre-operative chemotherapy, stratified by enterotoxigenic *Bacteroides fragilis* (ETBF) abundance (very low, low, high). (B) Pre-operative chemotherapy received: Overall-survival curve for patients who did receive pre-operative chemotherapy, stratified by the same ETBF categories. (C) Adjuvant chemotherapy none: Overall-survival curve for patients who underwent surgery without adjuvant chemotherapy, stratified by ETBF categories. (D) Adjuvant chemotherapy received: Overall-survival curve for patients who received adjuvant chemotherapy, stratified by ETBF status. *P* values represent log-rank tests; risk tables beneath each plot show the number of patients at risk at the indicated time points.
